# Roles of TRIM21/Ro52 in connective tissue disease-associated interstitial lung diseases

**DOI:** 10.3389/fimmu.2024.1435525

**Published:** 2024-08-06

**Authors:** Xiangmei Gong, Shukang He, Pengcheng Cai

**Affiliations:** Department of Clinical Laboratory, Union Hospital, Tongji Medical College, Huazhong University of Science and Technology, Wuhan, China

**Keywords:** TRIM21/Ro52, autoantibody, autoimmune disease, connective tissue disease, interstitial lung disease

## Abstract

Multiple factors contribute to the development of connective tissue diseases (CTD), often alongside a range of interstitial lung diseases (ILD), including Sjögren’s syndrome-associated ILD, systemic sclerosis-associated ILD, systemic lupus erythematosus-associated ILD, idiopathic inflammatory myositis-associated ILD. TRIM21(or Ro52), an E3 ubiquitin ligase, plays a vital role in managing innate and adaptive immunity, and maintaining cellular homeostasis, and is a focal target for autoantibodies in various rheumatic autoimmune diseases. However, the effectiveness of anti-TRIM21 antibodies in diagnosing CTD remains a matter of debate because of their non-specific nature. Recent studies indicate that TRIM21 and its autoantibody are involved in the pathogenesis of CTD-ILD and play an important role in diagnosis and prognosis. In this review, we focus on the contribution of TRIM21 in the pathogenesis of CTD-ILD, as well as the potential diagnostic value of its autoantibodies in different types of CTD-ILD for disease progression and potential as a novel therapeutic target.

## 1 Introduction

Connective tissue disease (CTD) is a group of autoimmune diseases that can involve multiple organ systems, among which interstitial lung diseases (ILD) are one of the common complications (1). ILD is a disease that affects the lung parenchyma, characterized by inflammation, fibrosis, and damage of the interstitium around the alveoli, including the alveolar walls and interstitial tissue (2). ILD can be associated with most types of CTD, including rheumatoid arthritis (RA), systemic lupus erythematosus (SLE), systemic sclerosis (SS), Sjogren’s syndrome (SjS), and mixed connective tissue disease (MCTD); but it can be associated with other factors, such as medications and infections (3, 4). The general treatments include anti-inflammatory drugs such as corticosteroids, immunomodulators, and other supportive measures (5). However, the mortality rate of CTD-ILD is extremely high, and its pathophysiology is still not well understood. The molecular biomarkers for screening CTD-ILD are also not yet clear, leading to ongoing diagnostic and classification issues. In addition, intrinsic differences in the pathogenic mechanisms of autoimmune disease-associated disorders are probably reflected in different patterns of inflammation and tissue damage, and subtypes of individual diseases are correlated, and other potential markers might exist for CTD-ILD. It is worth noting that research has found a possible association between the presence of anti-tripartite motif-containing 21 (TRIM21 or Ro52) antibodies and poor quality of life and high mortality in ILD.

**Figure 1:**
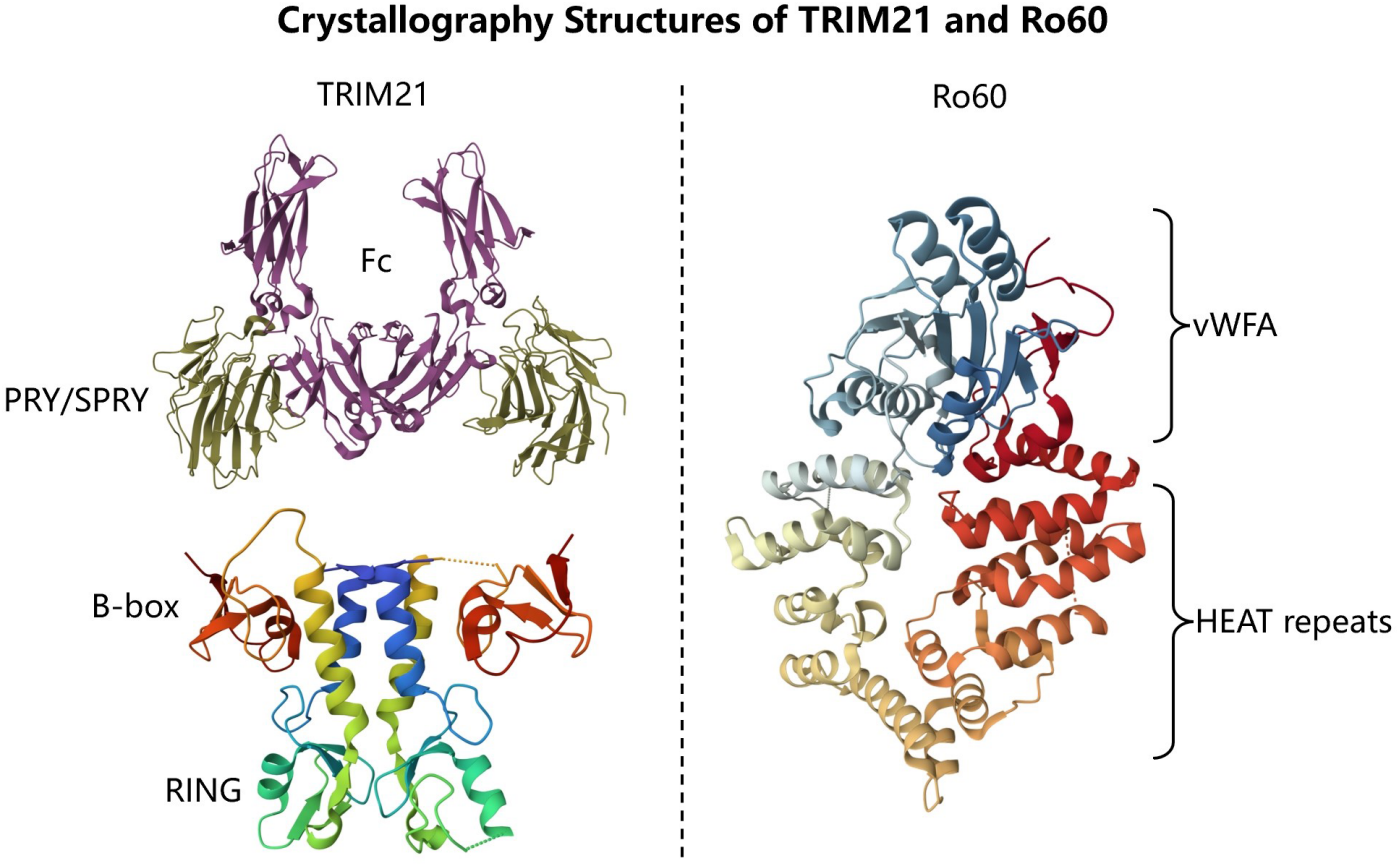
Crystallography Structures of TRIM21 and Ro60. TRIM21 consists of RING, B-box, coiled-coil and PRY/SPRY domains (PDB: 2IWG, 5OLM). Ro60 consists of a toroid of HEAT repeats and a von Willebrand factor A (vWFA) domain (PDB:1YVR).

TRIM21, an autoantigen detected in 1988 in the sera of patients with autoimmune diseases (6), is a 52-kD protein as an antibody-binding protein of unusual structure by yeast two-hybrid analysis, whose molecular structural characterization and encoding genes indicate that it has no homology to the Ro60 protein, although both are considered to be the main antigenic targets of anti-Ro/SSA antibodies **Figure 1** (7, 8). Autoantibodies are often considered to be a response to defects in the immune system that may result from excessive apoptosis or necrotic cell death, causing the immune system to mistakenly perceive its own cells and tissues as foreign invaders and mount an immune response against them (9). Generally, it is accepted that TRIM21 is involved in the occurrence and development of autoimmune diseases, and its autoantibodies have also been detected in patients with different types of autoimmune diseases (10). However, the significance of TRIM21 and its antibodies in the pathogenesis of CTD-ILD and their implications for the disease progression have not been fully elucidated in current studies. Therefore, this review will discuss the tissue and cellular expression distribution of TRIM21 and its antibodies in the pathophysiological process of CTD-ILD, as well as their impact on the diagnosis and prognosis of CTD-ILD.

## 2 TRIM21/Ro52

There are more than 80 known members of the TRIM family, the majority of which have E3 ligase activity, and TRIM21 is no exception (10, 11). TRIM21, as a member of the TRIM family, consists of N-terminal Really Interesting New Gene (RING), B-box, and a central coiled-coil (CC) domain involved in mediating TRIM21 dimerization, as well as a C-terminal PRY/SPRY domain, **Figure 1** (10–13). The functions of the four different domains predict the remarkable biological potential of TRIM21.

The RING domain of TRIM21 is mainly involved in mediating Lysine 63(K63) and Lysine 48(K48)-linked ubiquitination (14). K63 is involved in the regulation of DNA repair and inflammation signaling, while K48-modified proteins are involved in targeted protein degradation via the proteasome (14, 15). Studies have shown that the RING domain of TRIM21 binds with the ubiquitin-conjugating enzyme E2W (Ube2W), thereby mediating the ubiquitination of K63-linked protein complexes bound by TRIM21 (16). In peripheral blood mononuclear cells (PBMCs), macrophages, and dendritic cells (DCs), intracellular TRIM21 mediates the ubiquitination and degradation of key regulatory proteins in the STING signaling pathway through K48 linkage, thereby affecting the production of pro-inflammatory cytokines during disease progression (17–19). It is worth noting that the intermolecular dimerization of the RING domain is one of the important mechanisms of TRIM21 ubiquitination (20). However, inhibiting RING domain does not affect the ubiquitination and degradation of substrates mediated by TRIM21 (21).

The B-box domain, as another important regulatory factor of TRIM21, plays a crucial role through its interaction with the RING domain (22). Research has observed that the B-box domain can act as an E2 mimic, inhibiting the ubiquitination function of TRIM21 by occupying the E2 binding site on the RING domain; however, TRIM21 could counteract the inhibition of the B-box by phosphorylating inhibitory kappa B kinase β (IKKβ) and transforming growth factor β-activated kinase 1(TBK1) at the LxxIS motif on the RING domain (22–24). The CC domain has a hydrophobic core that contributes to stabilizing protein conformation and mediates interactions between proteins (25). Recent studies have shown that the CC domain promotes TRIM21 self-association into homodimers through dimerization, thereby enhancing the E3 ubiquitin ligase activity of TRIM21’s RING domain (20, 26, 27).

TRIM21 could bind to most immunoglobulins thanks to the complex tertiary structure of its C-terminal B30.2 domain (also termed PRYSPRY) (28). The PRY/SPRY domain of TRIM21 contains a high-affinity immunoglobulin Fc-binding site interacting with immunoglobulins with specificity and extremely high affinity, which is highly conserved by binding to the CH2-CH3 interface, and does not overlap with the Fc domains of Fc gamma receptors (FcγR) and C1q (28). Besides attaching to IgG, TRIM21 is also capable of binding to IgA and IgM, albeit with a markedly reduced affinity in comparison to IgG (29–31). Therefore, the high affinity between PRY/SPRY domain of TRIM21 and IgG may be associated with promoting the pathogenic accumulation of immune complexes formed by anti-TRIM21 autoantibodies in autoimmune diseases. Moreover, recent study showed that histone deacetylase 6 (HDAC6) promotes the homodimerization of TRIM21 through its PRY/SPRY domain, indicating that the PRY/SPRY domain regulates the homodimerization of TRIM21 through acetylation, thereby indirectly affecting the E3 ubiquitin ligase activity mediated by TRIM21 (32).

It is now widely recognized that TRIM21 is primarily present in the cytoplasm, where it regulates cytoplasmic substrates through ubiquitination (33). The localization of TRIM21 is regulated by endogenous sequences, in particular the leucine zipper in the domain of the convoluted helix structure, which has been proven to play an important role in the cytoplasmic localization of TRIM21 (34, 35) When this region is deleted, TRIM21 rapidly translocates to the nucleus. Previous studies have demonstrated that TRIM21 can interact with E2s Ube2D1 located in the cytoplasm and Ube2E1 located in the nucleus (36, 37). Moreover, inflammatory mediators such as nitric oxide (NO) and interferon (IFN)-α induce TRIM21 and contribute to TRIM21 translocation to the nucleus (38–40). Therefore, TRIM21 exerts a broad intracellular defense by translocation with the help of cellular compartmentalization for the regulation of the topological distribution of molecules that are harmful or desirable to pathogens.

## 3 Role of TRIM21/Ro52 in immune and inflammatory responses

### 3.1 TRIM21 in intracellular immunity

Activation of the nuclear transcription factor kappaB (NF-κB) and interferon regulatory factor (IRF3) pathways occurs through intracellular delivery of antibodies that bind to specific pathogens *in vivo* during infection, which enhances innate immune signaling (31, 41). In addition to the dependence of intracellular pathogens on the proteasome for their degradation in the cell, TRIM21 of the cytoplasmic antibody receptor can also be involved in exerting the effects of intracellular antibody-mediated pathogen degradation (41). Studies have reported that TRIM21 acts primarily on viral particles that enter the cytoplasm and are bound by specific antibodies (31). *In vitro* experiments revealed that antibody-dependent intracellular neutralization of TRIM21 up-regulated by IFN-α was enhanced in adenovirus-infected Hela cells, and viral infection was inhibited. However, effective virus clearance also needs to be dependent on the E3 ubiquitin ligase activity of TRIM21 and degradation involving the proteasome.

**Figure 2:**
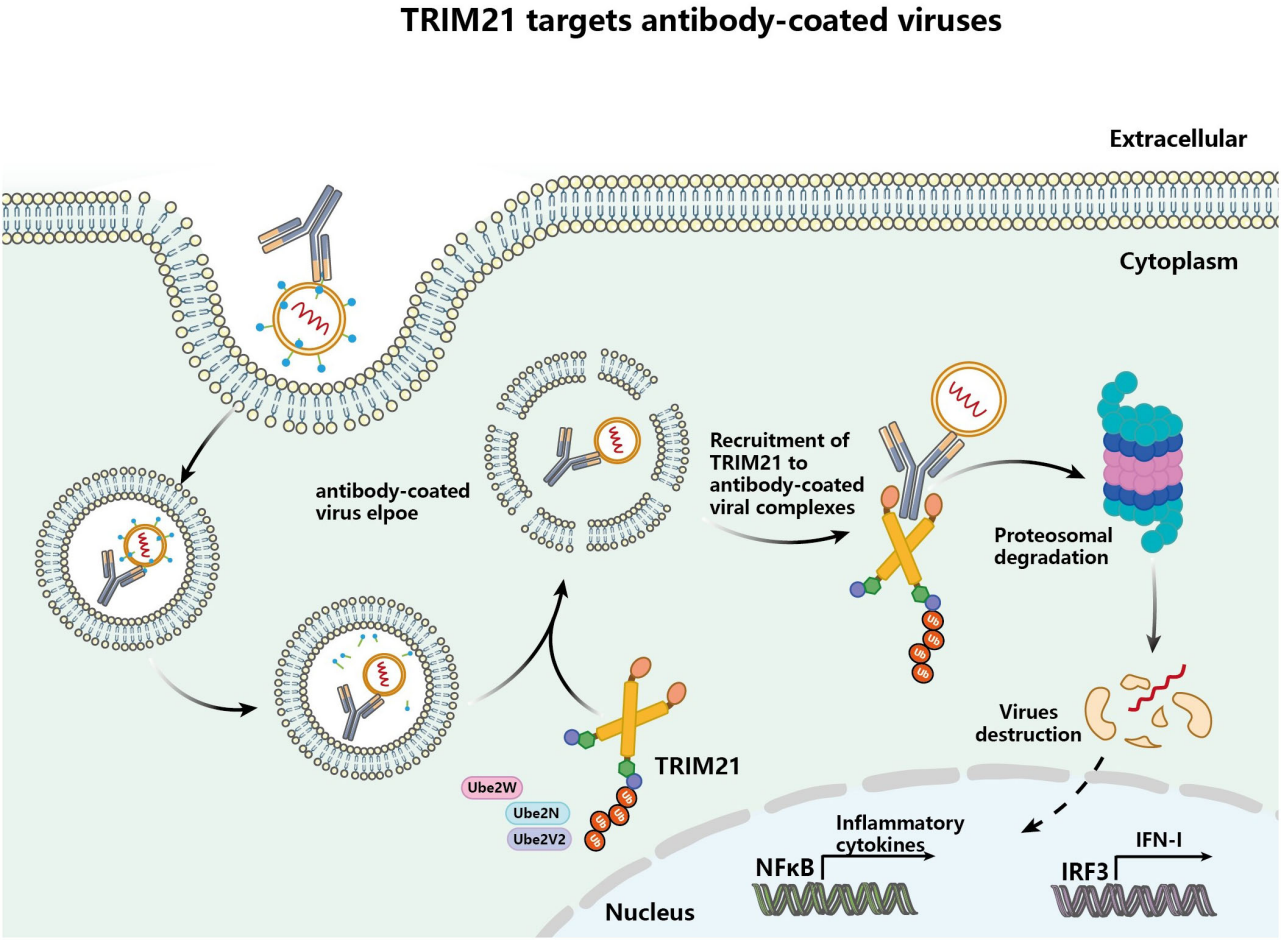
TRIM21 targets and destroys antibody-coated pathogens via ubiquitination and degradation (viruses as an example). Pathogens coated by antibodies are translocated into the cytoplasm, where the intracellular Fc receptor TRIM21 binds the antibody coating the pathogen and is activated to initiate ubiquitination and proteasome-dependent degradation of the targeted pathogen.

Consequently, TRIM21 is also limiting for the clearance of antibody-bound pathogens, involving multiple important cofactors (42). TRIM21 requires recruitment of the E2 enzyme Ube2W and catalytic auto-monoubiquitination before exerting its effects and recruits the E2 heterodimer Ube2N/2V2h and the deubiquitinase Poh1, which promotes elongation of K63 polyubiquitin chains, **Figure 2** (16, 43). Ubiquitinated TRIM21 serves as a substrate for the proteasome, recruiting proteasomes to facilitate the degradation of intracellular pathogens and thus, contributing to the initiation of innate immune signaling (16). In conclusion, in intracellular immunity, TRIM21 functions through antibody-dependent intracellular neutralization with antibody-bound pathogens associated with pathogens in infected cells, as well as intracellular antibody-mediated degradation of pathogens in conjunction with the proteasome.

**Figure 3:**
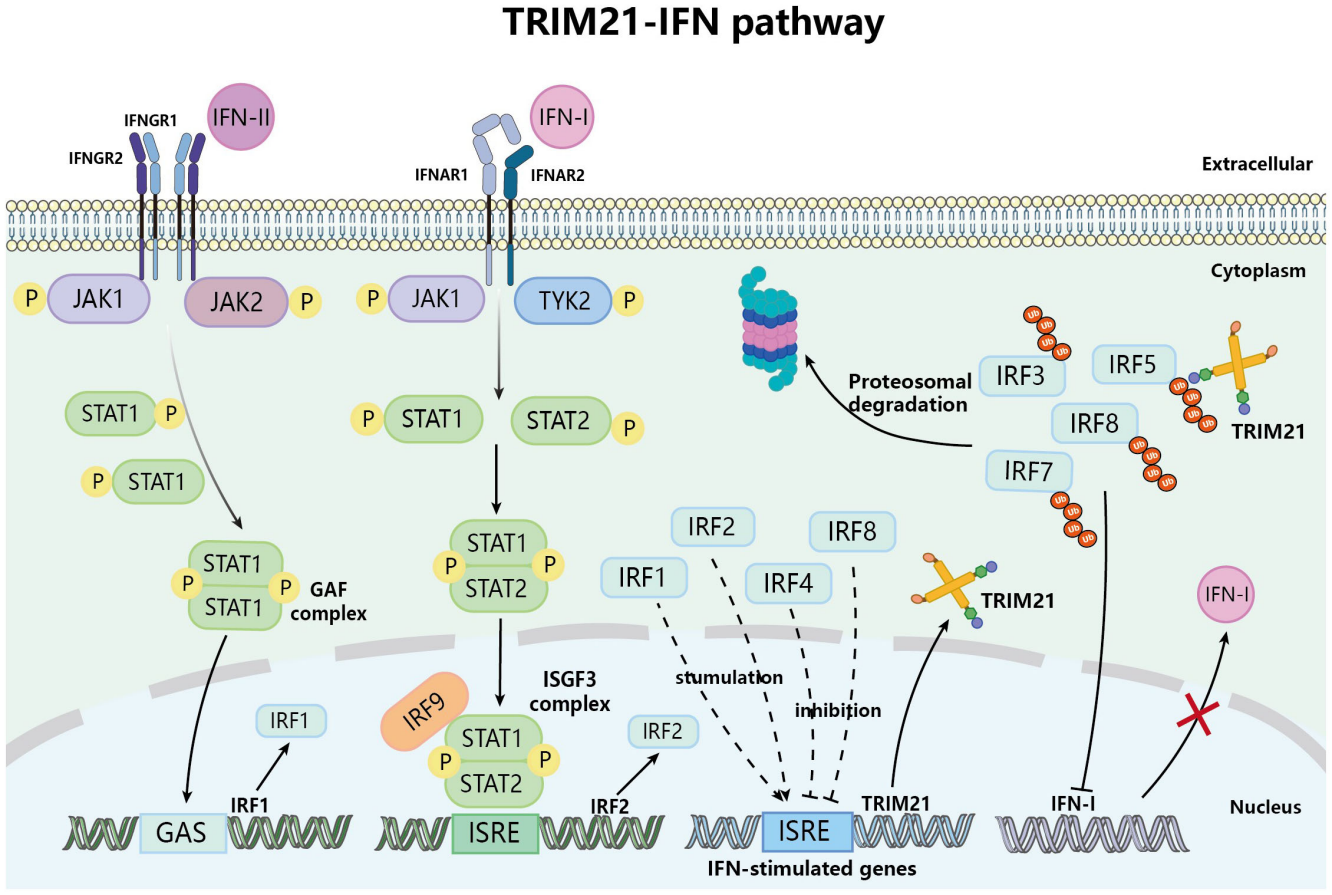
TRIM21 and IFN signaling pathway. Upon attaching to their specific interferon receptors, IFN-I and IFN-II facilitate the phosphorylation of the JAK/STAT pathway, resulting in the formation of ISGF3 and GAF complexes, respectively, initially triggering the expression of IRF1 and IRF2. IRF1 and IRF2 translocate to the nucleus and bind to ISRE, thereby inducing the expression of IFN-stimulated genes that include TRIM21, thus promoting the upregulation of TRIM21. However, the upregulated TRIM21 inhibits the expression of IFN-I by ubiquitinating IRF3/5/7/8.

Moreover, in turn, gene expression of TRIM21 is regulated by immune signaling. Basic research has found that IFN-I and IFN-II can induce TRIM21 mRNA expression in various cells such as Hela cells, EL4 cells, and macrophages (44–46). Research showed that after IFN-I binds to cell surface receptors and stimulates the phosphorylation of JAK1 and TYK2, it promotes the phosphorylation of downstream molecules STAT1 and STAT2, which then form the IFN-stimulated gene factor 3 (ISGF3) with IRF9, thereby inducing the expression of IRF2; on the other hand, IFN-II stimulates downstream signaling molecules to produce IRF1 through a signaling cascade, **Figure 3** (47). Both IRF1 and IRF2 can bind to the IFN-stimulated response elements (ISRE) site of the TRIM21 gene, thus upregulating the expression of TRIM21 (46). Of course, TRIM21 is expressed in various immune cells such as macrophages, DCs, neutrophils, B cells and T cells. TRIM21 influences disease outcomes and prognosis differently by regulating the expression of upstream and downstream molecules in immune cell signaling pathways and the production of cytokines in different immune cells.

### 3.2 TRIM21 and dysregulation of immune cell homeostasis

#### 3.2.1 Macrophages

**Figure 4:**
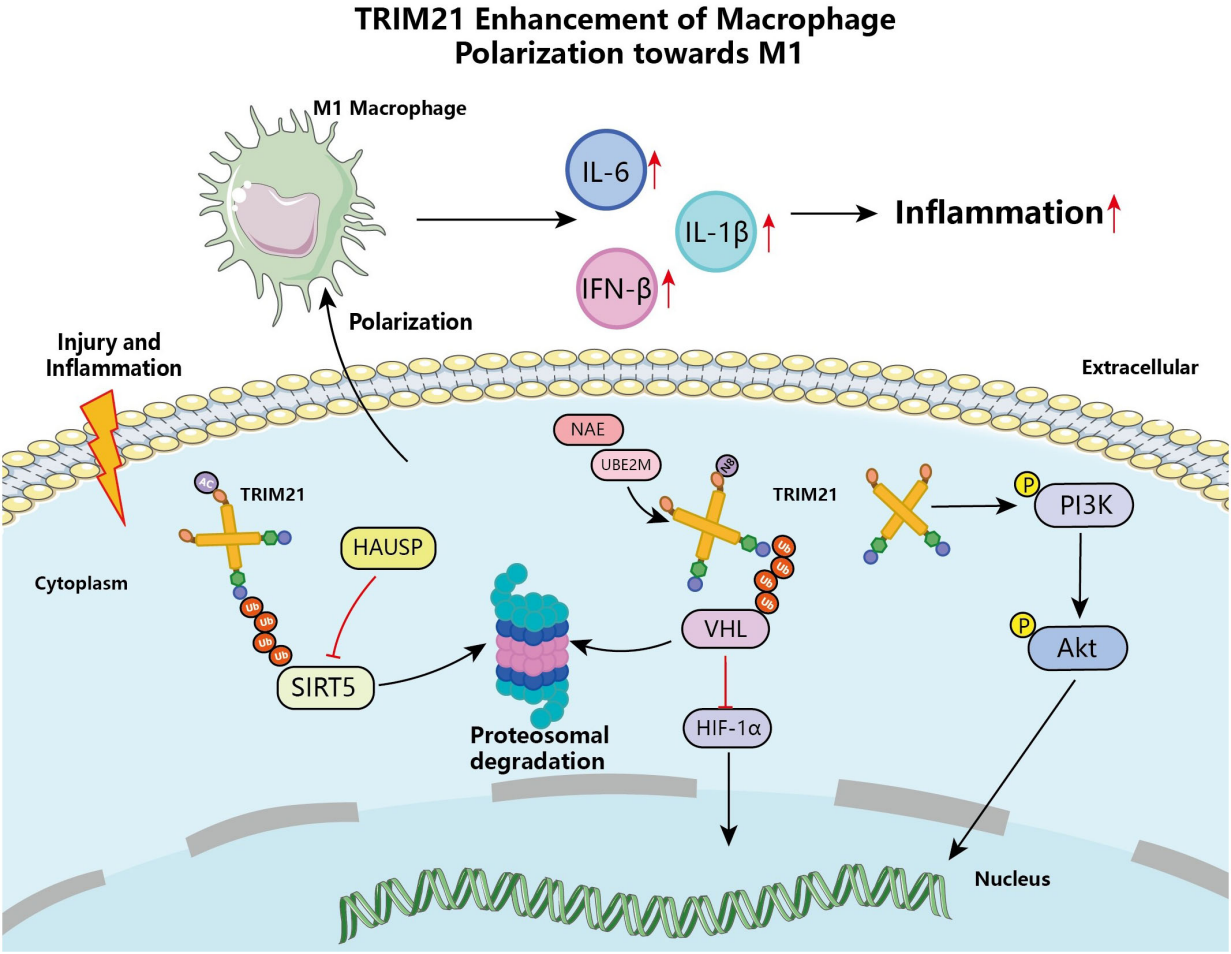
TRIM21 enhancement of macrophage polarization towards M1 macrophage. TRIM21-regulated signaling pathways are involved in macrophage polarization during injury or inflammation, involving the PI3K/Akt signaling pathway. Together with the proteasome, TRIM21 is involved in the degradation process of complexes of VHL-HIF-1α and SIRT5, which promotes the polarization of macrophages toward M1 macrophages, enhances the expression of pro-inflammatory cytokines, and exacerbates the inflammatory process.

Macrophages are effective immune effector cells that play a crucial role in both tissue homeostasis and injury, such as promoting tissue damage and progression, as well as facilitating wound healing and tissue remodeling in various pathogenic conditions (48). TRIM21 in macrophages targets different regulatory proteins, exhibiting dual roles in the immune system’s defense and response. Research has shown that compared to monocytes, macrophages significantly express higher levels of TRIM21, indicating that it may be an important regulatory factor in macrophages’ resistance to infectious and autoimmune diseases (49). *In vitro* experiments also have shown that human THP1-derived macrophages and mouse macrophages are activated and upregulate the expression of TRIM21 mRNA through Toll-like receptor 3 (TLR3) and TLR4 ligands (50). IFN-I signaling-activated STAT1 promoted TRIM21 expression in macrophages, leading to Ube2M, an E2 NEDD8-binding enzyme involved in neddylation (a post-translational modification analogous to ubiquitination), ubiquitinated by TRIM21 and degraded by the proteasome (51). Interestingly, macrophage Ube2M was involved in the induced neddylation of TRIM21, enhancing the interaction between TRIM21 and Von Hippel-Lindau (VHL), promoting TRIM21-dependent ubiquitination and degradation of VHL (52). This inhibited the proteasomal degradation of hypoxia-inducible factor-1α (HIF-1α), further inducing the production of IL-1β in macrophages, thereby exacerbating obesity-induced inflammation and metabolic disorders, **Figure 4**. Similarly, inhibiting Ube2M-mediated neddylation of TRIM21 in IL-17A^+^Foxp3^+^ Treg cells suppressed the expression of IL-1β, thereby reducing the activity of DEPTOR-mTOR axis-related protein molecules, and consequently alleviating lipid accumulation and inflammation in hepatocytes (53, 54). Furthermore, lipopolysaccharide (LPS) induced macrophage polarization and enhanced the interaction between TRIM21 and SIRT5, **Figure 4** (55). SIRT5, functioning as a desuccinylase, hinders the pro-inflammatory response mechanism of LPS-activated macrophages by impeding the ectopic movement of pyruvate kinase M2 (PKM) to the nucleus and augmenting its pyruvate kinase activity via deacetylation (56). However, TRIM21, which was upregulated in inflammatory macrophages, targeted SIRT5 by ubiquitination and degradation, thereby abrogating the role of SIRT5 in inhibiting the expression of the inflammatory factor IL-1β, **Figure 4** (55). Studies have found that in the myocardial infarction (MI) mouse model, TRIM21 affects macrophage polarization through the PI3K/AKT pathway, promoting the transformation of macrophages into the pro-inflammatory M1 type, further exacerbating post-infarction cardiac dysfunction, **Figure 4** (57). Conversely, TRPM2-dependent TRIM21 ubiquitination results in reduced production of reactive oxygen species (ROS) and inflammatory cytokines in macrophages, thereby promoting autophagy and apoptosis in inflammatory macrophages, which in turn reduces the pro-inflammatory activity of macrophages (58).

Overall, the above studies indicated that TRIM21 in macrophages primarily influences disease progression by targeting specific proteins required for macrophage polarization for ubiquitination and degradation, indirectly determining the fate of macrophages. Current researches mainly focus on the regulation of inflammatory macrophages by TRIM21, but its role in the later stages of disease regulation is not significant. This is related to the functional crosstalk between various molecules and TRIM21 in the later stages of the disease, but the specific mechanisms still require further exploration.

#### 3.2.2 Dendritic cells

TRIM21 not only regulates cell signaling in macrophages but also assumes a crucial role as a regulator in DCs, contributing to innate immune responses. DCs, which are responsible for delivering antigens, could trigger a more extensive immune response by taking in, processing, and displaying antigen-derived substances from pathogens to other immune cells (59). In the study of mDC, it has been found that TRIM21 mediates the ubiquitination and subsequent degradation of intracellular DDX41 (18). DDX41, as one of the members of the DEXDc helicase family, relies on the binding of pathogenic DNA by STING (the signaling adaptor of the cytosolic DNA sensor) to co-localize in the cytoplasm and initiate IFN-I and cytokine responses (60–62). Through the interaction between the PRY/SPRY domain and the Lys9 and Lys115 sites of the DDX41 DEADc domain, K48 subsequently mediates the ubiquitination and degradation of DDX41, thereby negatively regulating the expression of IFN-β and inhibiting the innate immune response of mDCs to intracellular dsDNA and DNA viruses (18). Additionally, it has been reported that in LPS/IFN-γ induced human moDCs, TRIM21 mediates the proteasomal degradation of IRF8, thereby promoting the production of IL-1β, IL-23, and IL-12 (63).

However, it has been reported that antibodies bound to pathogens enter cells as immune complexes, and antigens from immune complexes can further enhance the adaptive immune response to CD8^+^T cells by increasing affinity between Fc and TRIM21 to enhance antigen cross-presentation in DCs (64–66). CLEC-1 (C-type lectin) binds to conserved protein structures exposed during programmed cell death, promoting tumor progression by facilitating immunosuppressive tumor microenvironment and hindering DC from cross-presenting dead cell-associated antigens to CD8^+^T cells (67). However, Drouin et al. found that TRIM21 is an endogenous ligand of CLEC-1 and, as a self-antigen released by necrotic cells, induces necroptosis, but the Fc of CLEC-1 does not bind to the FcR of TRIM21 (67), yet this mechanism has not seen further explanation.

#### 3.2.3 T cells

Research has indicated that TRIM21 plays a key role in the production of IL-2 in T-cells triggered by CD28 and is essential in managing the growth and diversification of T-cells (68). The primary expression of TRIM21 was observed in CD4^+^T cells within normal intestinal mucosal tissues, with a reduction noted in inflammatory tissues during IBD (69). Inhibiting the ubiquitination of TRIM21 and the degradation of IRF3 in colitis induced by TRIM21^-/-^mice caused an increase in IL-17^+^CD4^+^ and IFN-γ^+^CD4^+^T cells, accompanied by an intensified immune reaction from CD4^+^T cells, culminating in heightened inflammation of the intestinal mucosa (33, 70, 71). Similarly, low TRIM21 expression promotes the differentiation of CD4^+^T cells into Th17 cells in atherosclerotic plaques and increases IL-17A expression, further exacerbating the formation of atherosclerotic plaques (72), implying that the balance of T cell differentiation appears to be dependent on the regulation of TRIM21. Conversely, TRIM21 binds to ubiquitinated NF-κB through K63 in CD3^+^T cells in Oral lichen planus (OLP) tissue, resulting in a heightened release of pro-inflammatory factor in T cells, subsequently triggering the NF-κB signaling pathway and exacerbating tissue damage (73, 74).

#### 3.2.4 B cells

**Figure 5:**
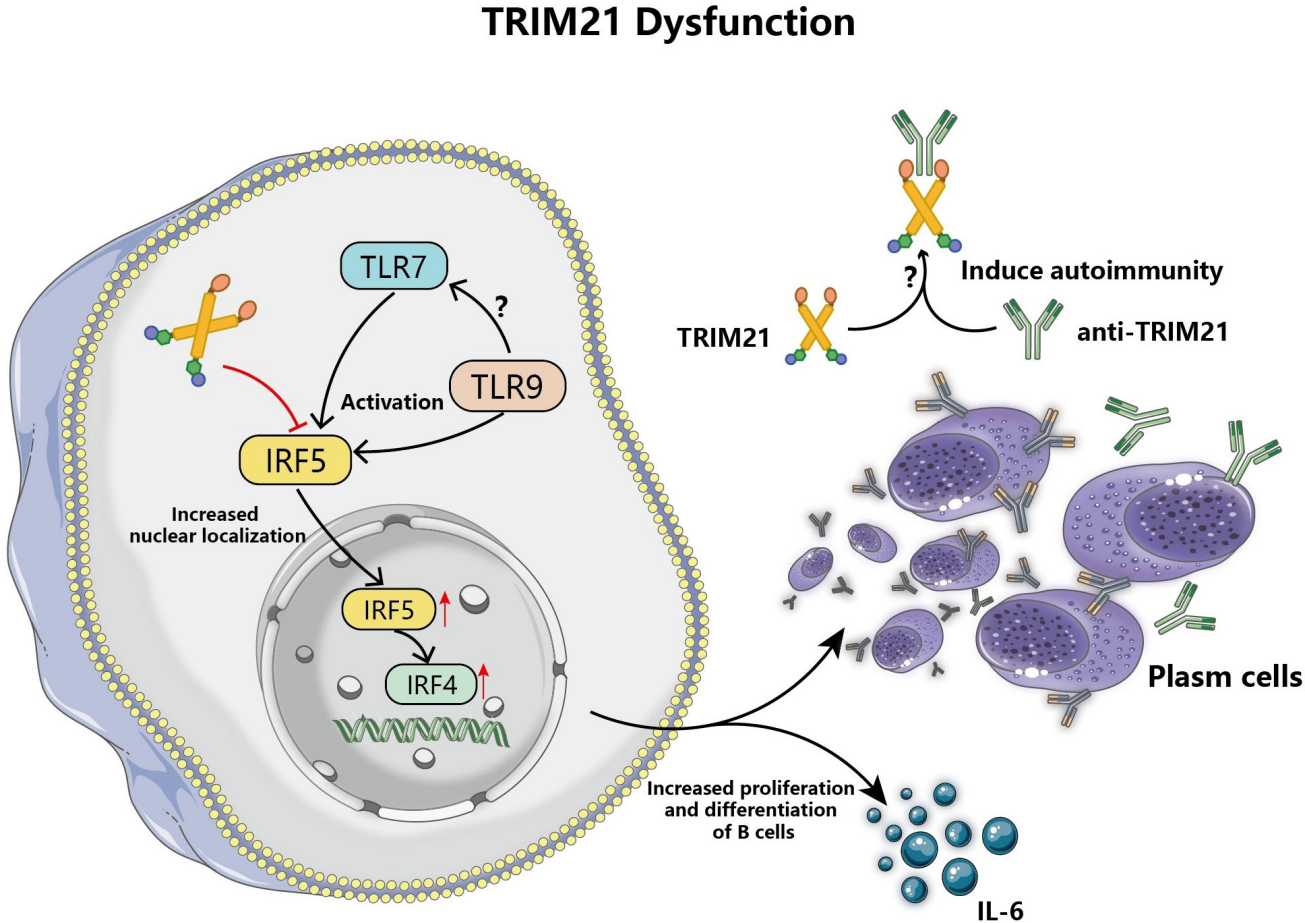
TRIM21 dysfunction leads to aberrant activation and proliferation of B cells. TRIM21 dysfunction attenuates the regulation of IRF5 ubiquitination, and TLR7 promotes IRF5 expression and nuclear localization in B cells to increase and enhance the expression of the downstream molecule, IRF4, and increase the differentiation of antibody-secreting plasma cells and the production of IL-6, which triggers autoimmune responses. In addition, TLR9 may balance the autoimmune damage caused by the abnormal proliferation of activated B cells stimulated by TLR7 through its regulatory function.

Additionally, TRIM21 dysfunction is involved in promoting aberrant activation and proliferation of B cells (75). Researches indicate that T cells and DCs contribute to the activation and diversification of B cells, but in lupus mouse models, the absence of TRIM21 did not alter the status of T cells and DCs, due to the abnormal function of B cells themselves (76, 77). TRIM21 participates in the regulation of B cells function by regulating the ubiquitination and degradation of IRF5, an important regulator of B cells differentiation and the production of antibodies by self-reactive B cells, **Figure 5** (78, 79). Overexpression of TRIM21 resulted in reduced IRF5 expression in B-cell lines during *in vitro* experiments, whereas the stimulation of B-cell growth lessened and encouraged apoptosis (79). Moreover, TLR7 enhances the nuclear localization of IRF5 in activated B cells and promotes the differentiation of B cells into plasma cells that secrete a variety of autoantibodies and thereby trigger tissue injury, among which TRIM21 has been found to be the target of secreted autoantibodies (80). TLR9 may affect B cell activation by indirectly regulating TLR7 through its signaling pathway, but the exact mechanism has not been elucidated, **Figure 5** (81). The relevant study also revealed that reduced TRIM21 gene expression in diffuse large B-cell lymphoma leads to increased aggressiveness of lymphoma and is associated with poor prognosis (75). However, the differentiation and proliferation of TRIM21 into adaptive immune cells vary due to elements such as triggers, tissue location, and the severity of inflammation, resulting in diverse disease outcomes.

Together, the proteins targeted by TRIM21 in different immune cells vary depending on the disease, indicating that TRIM21, as an E3 ubiquitin ligase, has extensive interactions and common regulatory characteristics within the immune system. As it’s been described, proteins in the signal transduction pathways designed in both innate and adaptive immune cells in diseases may be directly or indirectly regulated by TRIM21. However, due to the different functions of the proteins targeted by TRIM21, the impact on the disease progression also varies.

Therefore, understanding the general rules of how TRIM21 interacts with related pathway proteins in various immune cells within the immune system will help identify potential therapeutic targets in diseases. This requires further research and exploration.

## 4 TRIM21/Ro52 and connective tissue disease-associated interstitial lung diseases

The immune system is an intricate network of cells that distinguishes between self and foreign materials to efficiently combat invading pathogens (82). Nevertheless, malfunctioning peripheral and central immune tolerance mechanisms may cause immune cells to react to self-antigens, resulting in the widespread inflammation and tissue damage observed in autoimmune diseases (83). CTD is a common combination of autoimmune diseases, while ILD is one of the primary causes of morbidity and mortality among CTD patients. TRIM21 has been confirmed as the main target of autoantibody responses in CTD, and the value of anti-TRIM21 antibodies, as common autoantibodies in CTD, is increasingly being recognized in CTD-associated ILD. This section will focus on the potential diagnostic value of TRIM21 and its autoantibodies in the development of CTD-ILD based on currently published clinical research data and preclinical studies, as well as the possible molecular mechanisms involved in the pathogenic process.

### 4.1 TRIM21/Ro52 in Sjögren’s syndrome-associated interstitial lung disease

SjS is a systemic chronic inflammatory autoimmune disease that manifests as a decrease in the production of glands in the body, such as tears, saliva, sweat, and mucus, and the main symptoms may extend from dry mucosal surfaces to systemic involvement (84). Pulmonary involvement is a common extra-glandular complication, with ILD being the most common complication, with a prevalence of 10%-20% reported in studies (85, 86). Despite SjS-ILD typically exhibiting a mild and self-limiting progression, there was a notable rise in mortality cases among SjS patients with ILD (87, 88). Therefore, choosing suitable biomarkers is crucial for forecasting the variances between patients with advancing illness and those who might progress to a slow or stable phase of the disease.

In the 1960s, the serum of patients with SjS revealed the presence of anti-Ro/SSA antibodies, identified as a defining feature of SjS (89). While anti-TRIM21 antibodies frequently correlate with anti-Ro60, anti-TRIM21 positivity alone is found in 20-30% of SjS patients (90, 91). Research indicates that patients with high anti-TRIM21 titration experience more severe clinical symptoms, such as an enlarged parotid gland, a positive rheumatoid factor, and diminished blood cells. Similarly, patients with SjS exhibiting only anti-TRIM21 positivity showed elevated disease activity and a higher frequency of simultaneous mixed cryoglobulinemia, in contrast to those with anti-Ro60/La positivity or patients lacking these autoantibodies (92). In a cohort study of 527 patients with primary SjS in China, the researchers found that the detection rate of anti-TRIM21 positivity in patients with concomitant ILD was 36% and that anti-TRIM21 was independently associated with ILD, **Table 1** (95). In recent years other studies also have reported that up to 39-86% of patients with SjS-ILD exhibit anti-TRIM21 positivity, **Table 1** (96–103). Consistent with previous studies, the positivity of anti-SSB and anti-TRIM21 antibodies was significantly higher than in patients without ILD and may be predictive of the extensive lung involvement seen in patients with SjS-ILD (96, 98, 101, 102). However, in contrast, it has been suggested that there is no difference between SjS-ILD and SjS patients without ILD (SjS-NILD) in the presence of anti-SSA, anti-TRIM21, rheumatoid factor (RF), and antinuclear antibody (ANA) (100, 103). In conclusion, these cohort studies suggest that SS-ILD patients with anti-TRIM21 antibodies have more disease activity compared to patients without these autoantibodies, and that anti-TRIM21 is a possible cause of lung involvement leading to ILD.

**Table 1:** Main studies’ results regarding the prevalence of anti-TRIM21 antibody in SjS-ILD.

Reference	Country(cases)	Year	Prevalence in SjS	Prevalence in SjS-ILD	Findings
Chetrit ^1^(90)	USA (60)	1990	87%	N	In patients with SjS, the main Ro/SSA antibodies in serum are primarily anti-TRIM21, without concomitant anti-Ro60.
Locht ^1^(93)	Denmark (321)	2008	61%	N	The patients of SjS positive for anti-TRIM21, of which 56% had at least one organ manifestation.
Dugar ^1,2^(91)	Australia (40)	2010	97%	N	Anti-TRIM21 positivity is more prevalent compared to anti-Ro60; therefore, specific testing for their distinction in clinical settings is recommended. Anti-TRIM21 reactivity was significantly higher in SjS than SLE and associated with rheumatoid factor positivity.
Zhao ^1^(94)	China (110)	2013	60%	N	There were no independent associations of IL-33 levels with anti-TRIM21 and ANA antibodies in patients with SjS.
Dong (95)	China (527)	2018	29%	36%	ILD is the common pulmonary involvement of SjS and the prevalence of SjS-ILD is 39.1%. Logistic regression analysis showed that positive anti-SSA (OR 7.86) were relevant factors of SjS-ILD.
Shi (96)	China (158)	2020	30%	39%	Anti-TRIM21 (aOR=2.06 [1.14–7.65]), serum Angptl2 (aOR=4.13 [1.25–15.89]) and DLCO (aOR=9.51 [2.10–37.74]) were associated with SjS-ILD.
Zhang (97)	China (170)	2020	81%	86%	Patients of SjS with CT-UIP pattern had more male patients, older age of onset, and a lower frequency of anti-TRIM21 antibodies.
Xu ^3^(98)	China (113)	2020	N	48%	Extensive lung involvement is an independent risk factor for the progression of ILD in patients with SjS-ILD.
Zhao (99)	China (101)	2020	71%	76%	ILD was very common in SjS. ILD had a significant negative impact on the QoL of patients.
Guo (100)	China (735)	2021	73%	74%	SjS-ILD and SjS-NILD did not differ in the presence of anti-SSA, anti-SSB, anti-TRIM21, RF, anti-AMA-M2, anti-gp210, anti-dsDNA and anti-Sm antibodies, ANA.
Shi (101)	China (142)	2022	39%	56%	The prevalence of positivity for autoantibodies associated with pSS, such as anti-SSB and anti-TRIM21 but not anti-SSA, anti-ENA, or anti-Sm antibodies were significantly higher than patients without ILD.
Yang (102)	China (256)	2024	55%	64%	The positive rates of anti-TRIM21 antibodies were significantly higher in the SjS-ILD patients than in the SjS-NILD group.
Wang (103)	China (622)	2024	58%	53%	The anti-SSB positivity rates were lower in the SjS-ILD group than the SjS-NILD group (p=0.041), but ANA, anti-SSA, anti-TRIM21 and RF were similar between the two groups (all p>0.05).

^1^The report did not mention the cases of concurrent ILDs in SjS.

^2^Only the number of patients with diagnosis of SjS is shown in the Table; the number of patients with other disease diagnoses is not included.

^3^Only SjS-ILD patients were covered in the report.

anti-TRIM21, anti-tripartite motif-containing protein 21; SjS, Sjögren’s syndrome; SjS-ILD, Sjögren’s syndrome-associated Sjögren’s syndrome; SLE, systemic lupus erythematosus; anti-Sm, anti-Smith; ILD, interstitial lung disease; Angptl2, angiopoietin-like protein 2; DLCO, diffusion capacity of the lung for carbon monoxide; aOR, adjusted odds ratio; CT-UIP, computed tomography-usual interstitial pneumonia; QoL, quality of life; anti-SSB, anti-Sjögren’s syndrome related antigen B; anti-SSA, anti-Sjögren’s syndrome related antigen A; anti-ENA, anti-extractable nuclear antigen; SjS-NILD, Sjögren’s syndrome patients without interstitial lung disease; RF, rheumatoid factor; anti-dsDNA, anti-double-stranded deoxyribonucleic acid; ANA, antinuclear antibody.

Preclinical studies have shown that TRIM21 is significantly expressed in the ductal epithelium of focal tissues in salivary gland tissues of patients with primary SjS (pSjS), and the degree of TRIM21 expression in the ductal epithelium correlates with the degree of inflammation (104). Additionally, Aqrawi et al. suggested that Ro/SSA-specific memory B cells in the salivary glands of SjS patients are converted to plasma cells at the site of inflammation, thereby promoting autoantibody production (105). These data indicate that the overexpression of TRIM21 in the lesions of SjS patients is involved in the production of the specific autoantibody anti-TRIM21. Furthermore, during chronic inflammation, the overexpression of TRIM21 in secretory glands is involved in regulating immune cell infiltration, ultimately leading to tissue degeneration and salivary gland damage.

In SjS animal models, the passive transfer of TRIM21-positive serum from immunized mice to non-immunized model mice resulted in dysfunction of the salivary and lacrimal glands, along with local deposition of anti-TRIM21 antibodies in the glands (106), suggesting that anti-TRIM21 antibodies may be directly involved in the pathogenic process of SjS. Furthermore, in SjS, systemic B cell hyperactivity and high expression of IFN-I are the reasons for the elevated expression of TRIM21 on the surface of circulating plasmacytoid DCs (46, 107). Notably, TLR7-stimulated SjS salivary gland epithelial cells presented TRIM21 antigen via major histocompatibility complex (MHC) class I molecules (108). These results suggest that TRIM21 may function on the surface of immune cells to identify and destroy other immune cells.

Interestingly, patients with SjS appear to have autoantibody specificity for several different structural epitopes of the TRIM21 protein, including its RING, B-box, and CC domains (109). Sroka et al. demonstrated that anti-TRIM21 antibodies directed against this CC domain were involved in salivary gland dysfunction induced by E3 ubiquitin ligase activity and that IgG deposition in the glands was significantly increased (110). Moreover, *in vitro* experiments revealed that anti-TRIM21 antibodies from pSjS patients inhibited the E3 ligase activity of TRIM21 by binding to its RING domain (111). TRIM21 is widely expressed in all cells of the body, including those related to lung tissue. In patients with SjS associated with ILD, anti-TRIM21 antibodies appear to cause functional impairment of lung tissue through inhibition of TRIM21 ubiquitin ligase activity, leading to interstitial pneumonitis, but there are currently no reports on the mechanism of pathogenesis of SjS-ILD with TRIM21.

### 4.2 TRIM21/Ro52 in systemic sclerosis-associated interstitial lung disease

SSc is a rare autoimmune disease characterized by hyperplasia and hardening of connective tissue in the skin, internal organs, and blood vessels. Based on skin involvement levels, SSc is categorized into three types: limited cutaneous scleroderma, diffuse cutaneous scleroderma, and SSc sine scleroderma (112). Due to the limited treatment options, the advanced stages of SSc are often accompanied by life-threatening fibrotic complications, including SSc-ILD (113). The progression of SSc-ILD is believed to occur in three primary phases: first, abnormal activation of the immune system due to damage to the alveolar epithelium, endothelial cells, and vasculature, followed by abnormal activation of immune cells to release cytokines and autoantibodies, prompting fibroblasts to collect from lung and further promote its activation and adhesion, as well as the production of large amounts of extracellular matrix that eventually leads to normal pulmonary architecture being replaced by scar tissue (114).

Currently, autoantibodies have been identified in studies as specific serological biomarkers for SSc diagnosis and prognosis, including anti-centromere, anti-topoisomerase I (anti-Topo I), anti-RNA polymerase III, and two major types of SSA/Ro antibodies, anti-Ro60 and anti-TRIM21, which co-exist with them (115). Research indicates an increased likelihood of SSc-ILD in individuals with anti-Topo I, yet complications from ILD are rare in those with anti-centromere-positive SSc, despite anti-centromere being the most identifiable specific autoantibody in SSc, **Table 2** (124). In 2012, the Canadian Scleroderma Multicenter Study Group’s comprehensive analysis of autoantibodies associated with 963 patients with SSc showed that anti-TRIM21 was detected in 20% of patients and was the second most common autoantibody in the patient cohort, except for anti-centromere antibodies (119). Additionally, patients with SSc exhibiting anti-TRIM21 positivity demonstrated an increased probability of concurrent ILD (118, 119). In 2021, the Canadian scleroderma research team once more reported positive rate results for anti-TRIM21 in 26% of 1,698 SSc patients, marking the most extensive cohort analysis of anti-TRIM21 in SSc to date, and crucially, this aligns closely with the UK’s reported rate of anti-TRIM21 positivity in 1,010 SSc patients (27%), **Table 2** (117, 123). Interestingly, it was found that the probability of concurrent lung disease was relatively high among anti-TRIM21 positive patients relative to anti-TRIM21 negative patients, and there was an association with concurrent ILD (118, 125). For patients with SSc-ILD, the anti-TRIM21 antibodies were found to overlap with nearly all autoantibodies linked to SSc (119). Moreover, the levels of anti-TRIM21 were notably elevated in patients with SSc who tested positive for anti-Ro60 and anti-aminoacyl-tRNA synthetase antibodies (117). Additionally, the existence of anti-TRIM21 in patients with SSc-ILD was independently linked to lower survival rates (121). In conclusion, the data from the cohort studies robustly endorses both the function and predictive significance of anti-TRIM21 antibodies in diagnosing the condition in SSc-ILD.

**Table 2:** Main studies’ results regarding the prevalence of anti-TRIM21 antibody in SSc-ILD.

Reference	Country(cases)	Year	Prevalence in SSc	Prevalence in SSc-ILD	Findings
Fujimoto (116)	Japan (263)	1997	8%	8%	Anti-TRIM21 associated with concurrent SjS in SSc patients
Parker ^1^(117)	UK (1010)	2009	27%	N	Anti-TRIM21 is prevalent in populations with SSc. Anti-TRIM21 generally co-exist with anti-Ro60 or anti-synthetase antibodies in patients of SSc.
Mierau ^1^(118)	German (863)	2011	22%	N	Anti-Ro/anti-La, anti-mitochondrial, and anti-p25/p23 antibodies were most frequently detected in the cohort of 55.4% of the patients with SSc. Anti-Ro60, anti-centromere, and anti-Topo I had a strong association with pulmonary fibrosis. Anti-TRIM21 was not described with SSc-ILD in the cohort of the study.
Hudson (119)	Canada (963)	2012	20%	25%	Anti-TRIM21 were the second most common autoantibodies in this SSc. Anti-TRIM21 was strongly associated with ILD (OR=1.53 [1.11-2.12], P=0.0091) and overlap syndrome (OR=2.06 [1.01-4.19], P=0.0059).
Massie ^1^(120)	Canada (689)	2014	20%	N	There is no association between prolonged QTc and antiRo antibodies, anti-Ro60, or anti-TRIM21.
Wodkowski (121)	Canada (1574)	2015	27%	32%	Monospecific anti-TRIM21 were strongly associated with ILD and as an independent predictor of mortality in the large SSc cohort of 1574
Lee (122)	Australia (596)	2021	34%	46%	Anti-TRIM21 is independently associated with mortality in patients with SSc. Anti-TRIM21 positivity is an independent risk factor for the development of PAH and mortality but does not demonstrate any association with ILD or other surrogate measures of lung function in a large Australian cohort of SSc patients.
Hoa (123)	Canada (1698)	2022	26%	39%	Anti-centromere, or none of the tested autoantibodies (anti-TRIM21 included) had numerically lower risks of developing cancer within two years in the cohort of SSc. Anti-Topo I and anti-U1-RNP associated with cancer risk within two years of scleroderma onset.

^1^The report did not mention the cases of concurrent ILDs in SSc.

anti-TRIM21, anti-tripartite motif-containing protein 21; SSc, systemic sclerosis; SSc-ILD, systemic sclerosis-associated interstitial lung disease; anti-Topo I, anti–topoisomerase I; OR, odds ratio; QTc, QT interval corrected for heart rate; SjS, Sjögren’s syndrome; PAH, pulmonary arterial hypertension; ILD, interstitial lung disease; anti-U1-RNP, anti-U1 ribonucleoprotein.

Through immunohistochemical staining, studies have observed that TRIM21 is localized in alveolar M2 macrophages in the peripheral lung tissue of SSc-ILD patients and that the accumulation of anti-TRIM21 in bronchoalveolar lavage (BAL) fluid suggests a pathobiological link between TRIM21 and its autoantibodies and the progression of SSc-ILD (126). M2 macrophages are increased in both skin and peripheral blood in SSc patients, and despite the function of M2 in the disease process to inhibit the inflammatory process and participate in the repair of damaged tissues, SSc-ILD persists as a chronic inflammatory disease that continues to prolong, ultimately leading to persistent damage and irreversible dysfunction of lung tissue (127, 128). Recent study has found that TRIM21 targets Sohlh2 (a member of the basic helix-loop-helix transcription factor superfamily) for ubiquitination and degradation, inhibiting its high expression in M2 macrophages and its role in promoting macrophage polarization to the M2 phenotype (129). In the progression of SSc-ILD, TRIM21 may serve as a beneficial molecular regulator in the differentiation of macrophages into M1 and M2. However, the production of autoantibodies may impair the normal biological function of TRIM21, exacerbating disease progression. Therefore, TRIM21 may regulate the production of autoantibodies, and dysfunction of TRIM21 could lead to the advancement of autoimmunity.

B cells circulating in SSc-ILD patients were activated, resulting in an increased production of specific autoantibodies accumulating in the lung, causing localized tissue damage (130). Similarly, the tight-skin (TSK/+) mouse, used as an animal model for SSc, exhibited unusual B cell activation, heightened production of serum immunoglobulin, and increased autoantibody production (131, 132). These researches suggest that abnormally differentiated B cells might produce anti-TRIM21 antibodies, which could explain the presence of anti-TRIM21 detected in the serum of SSc-ILD patients. However, no study has yet reported the effect of TRIM21 and its autoantibodies in B-cell dysfunction on the SSc-ILD disease process in SSc animal models, and the possible mechanisms remain unclear.

### 4.3 TRIM21/Ro52 in systemic lupus erythematosus-associated interstitial lung disease

SLE, an autoimmune disorder impacting various systems and organs, leads to the development of diverse autoantibodies in patients, with clinical manifestations evolving from minor skin issues to critical organ failure (133). The lung is one of the common organs accumulated in SLE, and the main lung lesions are pleurisy, organizing pneumonia, pulmonary atrophy syndrome, occlusive fine bronchitis, pulmonary hypertension, small airway lesions, pulmonary embolism, and interstitial pneumonia (134, 135). ILD is a complication of SLE with an incidence of approximately 3-9% and is a key contributor to poor SLE prognosis (136). SLE-ILD might manifest at any phase of the illness, from its initial diagnosis to the disease’s 20-year progression, considering the patient’s age and the progression of concurrent diseases, with ILD being either clinically negligible in the early stages or exhibiting subclinical phases (137). Approximately 40-50% of SLE patients are positive for anti-TRIM21 antibodies, frequently combined with anti-Ro60 and anti-La antibodies; the occurrence of anti-TRIM21 antibody positivity by itself is uncommon in SLE, **Table 3** (141, 146, 147). In addition, Xia et al. found that the positive detection rate of anti-TRIM21 in SLE-ILD patients was 30%, but no correlation between SLE-ILD and anti-TRIM21 has been identified (143).

**Table 3:** Main studies’ results regarding the prevalence of anti-TRIM21 antibody in SLE-ILD.

Reference	Country(cases)	Year	Prevalence in SLE	Prevalence in SLE-ILD	Findings
Chetrit ^1^(90)	USA (90)	1990	47%	N	In patients with SLE, the main Ro/SSA antibodies in serum are primarily anti-Ro-60, without concomitant anti-TRIM21.
Yamamoto^1^ (138)	France (381)	2000	32%	N	In the SLE patients, 44% were positive for anti-U1-A RNP activity, 34% for anti-Sm-B, 44% for anti-SSA (32% for TRIM21 and 46% for Ro 60), 32% for anti-SSB/La, and 11% for anti-Ku reactivities.
Dugar ^1,2^(91)	Australia (67)	2010	56%	N	The mean signal intensity of anti-TRIM21 correlated with anti-Ro60 and anti-La in SjS and SLE.
Bruner ^1,2,3^(139)	USA (1540)	2012	N	N	Familial aggregation of anti-Sm/RNP, anti-P, and anti-Ro60 in SLE patient sibling pairs was observed. SLE patients of Simplex-pedigree had a greater positivity of anti-dsDNA (P = 0.0003) and anti-chromatin (P = 0.005) compared to patients with a multiplex SLE pedigree.
Jeon ^1^(140)	Korea (1052)	2013	42%	N	In systemic autoimmune diseases and their various IIF patterns, both anti-TRIM21 and anti-SSA were the most prevalent autoantibodies.
Menéndez ^1^(141)	(141)	2013	27%	N	Anti-Ro60 and anti-TRIM21 showed both common and specific associations in SLE.
Rastin ^1^(142)	Iran (98)	2017	37%	N	Among SLE patients, the prevalence of anti-SSA and anti-Ro52 antibodies is lower in males compared to females, and no male patients have anti-SSB antibodies.
Xia (143)	China (60)	2021	38%	30%	NSIP is the most common manifestation of SLE-ILD.
Tan ^1^(144)	China (161)	2021	47%	N	The positive anti-TRIM21 antibody (OR=15.926, P<0.05) was an independent risk factor for pulmonary inflammatory lesions in SLE patients with hematologic involvement.
Amezcua-Guerra ^1,2^(145)	Mexico (143)	2023	24%	N	Anti-TRIM21 and disease activity or organ-specific involvement showed no association when only SLE patients were included.

^1^The report did not mention the cases of concurrent ILDs in SLE.

^2^Only the number of patients with the diagnosis of SLE is shown in the Table; the number of patients with other disease diagnoses is not included.

^3^The report solely documented cases of SLE-ILD.

anti-TRIM21, anti-tripartite motif-containing protein 21; SLE, systemic lupus erythematosus; SLE-ILD, systemic lupus erythematosus-associated interstitial lung disease; anti-SSB, anti-Sjögren’s syndrome related antigen B; anti-SSA, anti-Sjögren’s syndrome related antigen A; anti-Sm/RNP, anti-Smith/ribonucleoprotein; anti-P, anti-ribosomal P; anti-dsDNA, anti-double-stranded deoxyribonucleic acid; IIF, indirect immunofluorescence; SjS, Sjögren’s syndrome; NSIP, nonspecific interstitial pneumonia; ILD, interstitial lung disease; OR, odds ratio.

Recent studies have found that IFN-I plays an important role in SLE pathogenesis, especially IFN-α (148). Studies have shown increased IFN-α expression and higher TRIM21 expression levels in PBMC from SLE patients compared to healthy controls (149). Animal model studies revealed that TRIM21^-/-^ mice developed SLE-related clinical manifestations after skin lesions and abnormalities in the IL-23-Th17 pathway, which triggered tissue inflammation and systemic autoimmunity, suggesting that TRIM21 protects the organism by negatively regulating pro-inflammatory factor production in SLE (33). IRFs can be ubiquitinated by TRIM21 through E3 ubiquitination of ligase activity in its RING domain, thereby affecting IFN-I (33, 39, 150). In SLE, anti-TRIM21 antibodies inhibit the degradation of IRF, leading to an imbalance in IFN expression and an increase in TRIM21 expression; however, this is not the case in anti-TRIM21 antibodies negative populations, suggesting that anti-TRIM21 antibodies are involved in the progression of anti-TRIM21 positive SLE by interfering with the ubiquitination and degradation of IRF by TRIM21 (149). In MRL/lpr lupus susceptible mice, a dysfunction in TRIM21 was associated with the pathological progression of SLE, atypical B cell differentiation, elevated autoantibody levels, and proteinuria (151). Additionally, the research also revealed that circulating B cells derived from patients with TRIM21-positive SLE had increased potential to differentiate into plasmablast compared to healthy controls (151). In conclusion, preclinical studies indicate that the dysfunction of TRIM21 in SLE appears to be due to the immune system mistakenly recognizing the TRIM21 antigen as a foreign substance, thereby breaking immune tolerance.

The connection between anti-TRIM21 antibodies and SLE-ILD is still not well understood in the current study among a limited group of SLE-ILD patients, but anti-TRIM21 antibodies positivity in SLE-ILD is greatly likely to be associated with negative patient outcomes, and the specific mechanism needs to be further explored.

### 4.4 TRIM21/Ro52 in idiopathic inflammatory myositis-associated interstitial lung disease

Idiopathic inflammatory myopathy (IIM) manifests as muscular feebleness, inflammation, and discomfort (152). The primary identified variants of IIM, as per clinical and serological characteristics, include polymyositis (PM), dermatomyositis (DM), and inclusion body myositis (IBM). During the diagnosis of IIM, autoantibodies are deemed dependable for distinguishing various myopathy subtypes, primarily divided into myositis-specific autoantibodies (MSA) and myositis-associated autoantibodies (MAA) (153). MAA frequently appears in cases of myositis overlap syndrome, yet it’s a group of antibodies linked to the disease and lacks specificity, including anti-TRIM21, which is frequently linked to a heightened risk of concurrent ILD (153). It’s estimated that more than 40% of patients globally suffer from concurrent ILD in IIM, with regional differences in prevalence, peaking at 50% in Asia and 23% and 26% in North America and Europe, respectively (154). Clinical data collected from researches showed that anti-TRIM21 antibodies are one of the independent serological risk factors for different subtypes of IIM-ILD, with a positivity rate of up to 50%, which is similar to the frequency of anti-Jo-1 in IIM-ILD and are associated with the severity and prognosis of IIM-ILD, **Table 4** (159, 161, 162, 164, 165, 168, 169). Within the PM subtypes, anti-synthetase syndrome (ASS) stands out, with anti-TRIM21 showing a greater likelihood of occurrence in ASS compared to other subtypes and frequently linked to the severity of the disease. It has been reported that patients with anti-TRIM21 positive ASS who are anti-Jo-1 and/or anti-PL7 positive have a significantly increased risk of concomitant ILD and exhibit more severe myositis and lower survival rates (170, 171). Similarly, patients with anti-MDA5 and anti-TRIM21 positive DM also tend to complicate more severe ILD and have a worse prognosis; Sabbagh et al. also concluded that anti-TRIM21 can be a predictive marker for complication of ILD in patients with juvenile myositis, and also emphasized that it is likely to be associated with the severity of the disease, a worse prognosis, and an increase in the dosage of immunosuppressant medications during the follow-up period (172, 173). In clinical practice, the main tools commonly used for ILD screening are pulmonary function tests (PETs). Vojinovic et al. and Gui et al. did not find progression of IIM-ILD and changes in PFTs over time in anti-TRIM21-positive patients with IIM, but found an increase in the patient’s lung diffusion capacity (DLCO) at five-year follow-up (162, 165). These clinical research data indicate that anti-TRIM21 antibodies seem to exist in IIM-ILD patients before any clinical symptoms appear, and they can be detected in the serum possibly even before IIM complicates with ILD, or even earlier. Therefore, the detection of anti-TRIM21 antibodies in IIM is of great significance for early prediction of the risk of concurrent ILD.

**Table 4:** Main studies’ results regarding the prevalence of anti-TRIM21 antibody in IIM-ILD.

Reference	Country(cases)	Year	Prevalence in IIM	Prevalence in IIM-ILD	Findings
Rutjes ^1^(155)	Netherlands (112)	1997	20%	N	It was initially reported that anti-TRIM21 and anti-Jo-1 often appear simultaneously in IIM, with a high positive rate.
Frank ^1^(156)	USA& UK (216)	1999	43%	N	Anti-TRIM21 are associated with myositis autoantibodies anti-Jo-1, and accompany with positive for anti-amyl-tRNA synthetase antibody, anti-SRP or anti-PM-Scl antibody.
Brouwer ^1^(157)	European countries (417)	2001	25% PM (27%), DM (24%), IBM (21%)	N	Anti-TRIM21 has strong association with anti-Jo-1 in IIM, and often together with other anti-synthetase.
Koenig ^1^(158)	Canada&USA (100)	2007	30%	N	Anti-TRIM21, anti-Ku, and anti-synthetases are most common autoantibodies in IIM.
Dugar ^1^(91)	Australia (147)	2010	24%, PM (24%), DM (40%), IBM (13%)	N	Anti-TRIM21 is not disease specific but may be of importance in patients with IIM.
Srivastava (159)	India (124)	2016	36%, PM (44%), DM (25%), JDM (23%), CTDM (68%)	75%	Patients with anti-TRIM21 had a significantly higher probability of combined ILD compared to anti-TRIM21-negative patients.
Infantino ^1^(160)	Italy (51)	2017	41%	N	Anti-TRIM21 were the most prevalent autoantibodies in IIM, frequently associated with anti-Jo1 antibodies.
Huang (161)	China (56)	2020	46%	71%	Anti-TRIM21 and anti-Jo-1 antibodies were independent serological risk factors for IIM-ILD.
Vojinovic (162)	Italy (165)	2021	33%	57%	Anti-TRIM21^+^ ILD patients showed a significant increase of DLCO at 1 and 5 years of follow-up. Anti-TRIM21 antibody and dyspnea at onset in IIM could predicted the development of ILD.
Bello ^1^(163)	USA (210)	2021	19%	N	Anti-TRIM21 and anti-PM-Scl75 are common MAAs in IIM.
Liang ^2^(164)	China (151)	2021	N	53%	Anti-TRIM21 is a relevant antibody for IIM.
Gui ^2^(165)	China (267)	2022	N	55%, PM-ILD (58%), DM-ILD (57%), CADM-ILD (51%)	Anti-TRIM21 antibodies were more frequent with anti-MDA5 and anti-Jo1 antibodies than in those with other MSAs.
Ghirardello ^1^(166)	Italy (411)	2023	26%	N	The specificity of individual anti-TRIM21 was 79%.
Cheng ^1^(167)	China (66)	2023	59%	N	The cluster 1 of patients, which had a moderate risk of infection, had a high probability of positive mechanic’s hands, periungual erythema, anti-TRIM21 antibody, and anti-Jo1 antibody.
Fang (168)	China (126)	2024	61%	78%	Anti-TRIM21 positivity (HR=0.437, 95% CI 0.199-0.960) are risk factors for ILD progression in patients with IIM.
Valle (169)	USA (94)	2024	42%	64%	Anti-Ro52 likely predicts more severe IIM-ILD compared to anti-SSA/SSB.

^1^The report did not mention the cases of concurrent ILDs in IIM.

^2^The report solely documented cases of IIM-ILD.

anti-TRIM21, anti-tripartite motif-containing protein 21; IIM, idiopathic inflammatory myopathy; IIM-ILD, idiopathic inflammatory myopathy- associated interstitial lung disease; anti-Jo-1, anti-histidyl-tRNA synthetase; anti-SRP, anti-signal recognition particle; anti-PM-Scl, anti-polymyositis-topoisomerase I; PM, polymyositis; DM, dermatomyositis; IBM, inclusion body myositis; JDM, juvenile dermatomyositis; CTDM, connective tissue disease associated myositis; ILD, interstitial lung disease; DLCO, diffusion capacity of the lung for carbon monoxide; anti-PM-Scl75, anti-polymyositis-topoisomerase I 75; MAA, myositis-associated antibodies; anti-MDA5, anti-melanoma differentiation-associated gene 5; PM-ILD, polymyositis-associated interstitial lung disease; DM-ILD, dermatomyositis-associated interstitial lung disease; CADM-ILD, clinically amyopathic dermatomyositis-associated interstitial lung disease; MSA, myositis-specific antibodies; HR, hazard ratio; CI, confidence interval; anti-SSA/SSB, anti-Sjögren’s syndrome related antigen A/Sjögren’s syndrome related antigen B.

Interestingly, in IIM-ILD, the presence of autoantibodies to TRIM21 disrupts immune homeostasis and is associated with poor prognosis, and the role played by TRIM21 antigens in the pathogenesis of IIM-ILD is also thought-provoking. TRIM21 expression was reduced in monocytes and CD4^+^T lymphocytes from IIM patients compared to healthy controls and resulted in decreased secretion of IL-6 and IFN-α from monocytes and IL-17 and TNF-α from CD4^+^T cells (17). Reports indicate that TRIM21 has the capability to suppress its E3 ligase function by engaging in anti-TRIM21 interactions with E2/E3 (111). However, Martín et al. showed no correlation between TRIM21 expression and anti-TRIM21 in IIM (17). Hassan et al. found that myositis patients with positive of anti-TRIM21 antibodies have an imbalance in TNF and IL-10 expression in serum (174). These findings may predict that during the pathogenesis of IIM, the reduced expression of TRIM21 weakens the ubiquitination process of related regulatory factors (such as IRFs), leading to an imbalance between pro-inflammatory and anti-inflammatory cytokines, thereby triggering a persistent chronic inflammatory process. Additionally, in muscle biopsy specimens from IIM patients positive for anti-TRIM21, the number of B cells increases, and the expression of BAFF (an important cytokine involved in B cell differentiation and maturation) and its receptors (BAFF-R, BCMA, and TACI) is upregulated, ultimately leading to the production of autoantibodies and the destruction of muscle tissue (175, 176).

Based on the existing clinical research data and the very limited preclinical study results, the detection of anti-TRIM21 antibodies appears to be a potential biological marker for detecting the occurrence, development, and prognosis of IIM-ILD. Additionally, the expression of TRIM21 in muscle tissue and the dysfunction of its E3 ubiquitin ligase significantly affect the disease outcomes of IIM-ILD. However, the specific molecular mechanisms require further research exploration.

### 4.5 TRIM21/Ro52 in rheumatoid arthritis-associated interstitial lung disease

ILD is a common extra-articular manifestation in patients with RA, often worsens the prognosis of the disease, and leads to considerable illness and increased death rates in those with RA (177). Research indicates that the occurrence of RA-ILD varies between 10% and 61% (178). Multiple risk elements contribute to RA-ILD, encompassing advanced age, active synovitis, seropositivity for RF and anti-cyclic citrullinated peptide (anti-CCP), along with MUC5B polymorphism. RA-ILD is mostly asymptomatic in its early stages, making early diagnosis difficult. Clinically, lung involvement in RA-ILD patients is often discovered after joint damage has occurred, but it is worth noting that the lungs may already be affected in the early stages of RA, remaining in a state of mild chronic inflammation. Paulin et al. predicted aberrant activation of myofibroblasts and alveolar epithelial cells in the lungs early in RA-ILD, initiation of a systemic immune response at the level of the pulmonary mucosa by the process of citrullination, and subsequent migration of anti-CCP antibodies from the lungs to the synovium, which exacerbates joint damage (179).

It is noteworthy that studies have reported a lower positivity rate of anti-TRIM21 in RA compared to other CTD, **Table 5** (180, 182). Nonetheless, RA patients lacking RA-related risk factors may continue to experience concurrent ILD (178). Pertinent studies have reported that among females with preparatory RA, RF-positive patients were more positive for anti-TRIM21 than RF-negative patients, and both were concurrently positive for anti-Ro60 (183). Recently, researchers have proposed another possible pathway: the immune response of RA-ILD is located in the synovial tissue, where Th cells produce specific cytokines that spread the inflammation to the lungs, causing fibroblasts to differentiate into myofibroblasts, ultimately leading to ILD (179). Moreover, current studies have shown that lung tissue macrophages express TRIM21 and increased TRIM21 expression in monocytes has been found to promote Th1 and Th17 differentiation in other diseases, which seems to predict that TRIM21 positively regulates the disease process of RA-ILD in the course of RA-ILD, but the specific mechanism has not yet been reported in any study (126, 184).

**Table 5:** Main studies’ results regarding the prevalence of anti-TRIM21 antibody in RA-ILD.

Reference	Country(cases)	Year	Prevalence in RA	Prevalence in RA-ILD	Findings
Ricchiuti ^1,2^(180)	USA& UK& France&Poland (70)	1994	14%	N	The presence of Anti-TRIM21 was mainly noted in SjS, with a lesser occurrence in SLE, RA, JCA, and MCTD.
Dugar ^1,2^(91)	Australia (19)	2010	21%	N	–
Almagro ^1,2^(181)	Spain (16)	2016	5%	N	–
Meek ^1,2^(182)	The Netherlands (16)	2018	13%	N	Nearly all SjS patients were positive for anti-TRIM21 and anti-Ro-60, regardless of EGM, in contrast to RA patients where these antibodies were not present.

^1^The report did not mention the cases of concurrent ILDs in RA.

^2^Only the number of patients with the diagnosis of RA is shown in the Table; the number of patients with other disease diagnoses is not included.

anti-TRIM21, anti-tripartite motif-containing protein 21; RA, rheumatoid arthritis; RA-ILD, rheumatoid arthritis-associated interstitial lung disease; SjS, Sjögren’s syndrome; SLE, systemic lupus erythematosus; JCA, juvenile chronic arthritis; MCTD, mixed connective tissue disease; EGM, extraglandular manifestations.

RA patients with anti-TRIM21 positivity often have concurrent with other subtypes of CTD and ILD. The presence of anti-TRIM21 antibodies may indicate the occurrence and progression of ILD, but the TRIM21 antigen may only indirectly influence RA in the context of other concurrent CTD (185). However, there are currently no reports on TRIM21 in RA and RA-ILD, and the diagnostic efficacy of anti-TRIM21 in diagnosing RA-ILD remains controversial. The molecular mechanisms executed by TRIM21 in RA-ILD are still a mystery.

## 5 Conclusions and future directions

Differences in the structural domains and functions with which TRIM21 interacts in immune activity give it multiple roles in the immune response. TRIM21, as an intracellular receptor whose PRY/SPRY domain binds to the Fc region of the antibody complex, is of great significance for the intracellular detection of pathogens that have escaped neutralization by extracellular antibodies and have entered into the host cell. Subsequently, TRIM21 is involved in the regulation of immune signaling through its E3 ubiquitin ligase activity in the ubiquitination and degradation of key proteins and pathogen complexes within immune cells.

Differences in the regulation of different molecules by TRIM21 lead to its high requirement for target specificity in different immune cells. Variations in how TRIM21 regulates distinct molecules during inflammation result in it displaying contrasting immune responses, either anti-inflammatory or pro-inflammatory, across various infectious diseases. It’s a recognized fact that increased levels of IFN enhance TRIM21 expression, yet, paradoxically, heightened TRIM21 levels lead to the ubiquitination of IRF, thereby attenuating the immune effects produced by IFN. Therefore, TRIM21 may act as a lever to maintain and regulate immune homeostasis through interactions with endogenous proteins, though its underlying mechanisms still require further investigation. Additionally, whether TRIM21’s interactions with different signaling proteins play a decisive role in the maturation and differentiation of immune cells also needs more demonstration.

**Figure 6:**
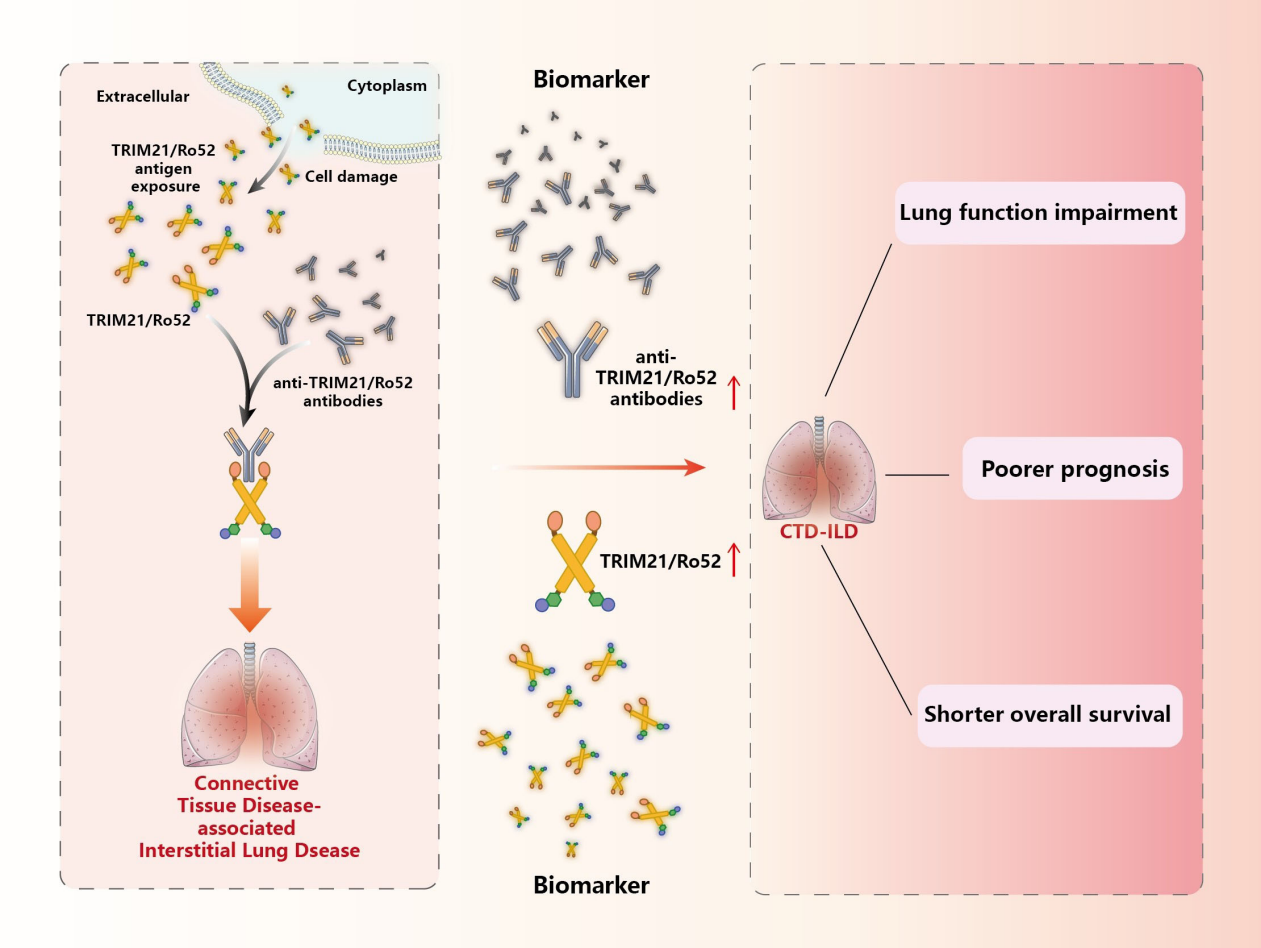
TRIM21/Ro52 and autoantibodies with CTD-ILD. TRIM21/Ro52, as the antigen targeted by specific anti-TRIM21/Ro52 antibodies, exists as an antigen that disrupts innate immunity, triggering CTD-associated ILD. TRIM21/Ro52 and anti-TRIM21/Ro52 antibodies, as potential biomarkers for CTD-ILD, may be related to lung tissue and functional damage, poorer prognosis, and shorter survival in CTD-ILD patients.

Dysfunctions in TRIM21 structure or function have been associated with autoimmune imbalances in the body. Mutated TRIM21 may be recognized by the immune system as an intracellular antigen and produce anti-TRIM21 antibodies, but it is not known whether TRIM21 can be located on the cell surface. In addition, impairment of TRIM21 ubiquitination activity may also lead to the production of anti-TRIM21 antibodies, which may result in relevant cellular dysfunction in the body, followed by apoptosis that exposes TRIM21 to the cell surface and induces a stronger immune response leading to autoimmune diseases such as CTD-ILD, (**Figure 6**).

TRIM21 is highly expressed in lungs compared to other tissues due to its high antigenicity (186). Additionally, viral infections are a trigger for various types of CTD, and TRIM21, as a cytosolic receptor for IgG antibodies, plays a crucial role in combating viral infections (187). Therefore, more preclinical studies are needed to explore whether TRIM21 and its autoantibodies accumulate in target organs before the onset of CTD-ILD and why they only affect specific tissue organs. In addition, the fact that TRIM21 maintains the anti-inflammatory properties of lung endothelial cells may be partial evidence for the existence of a correlation between anti-TRIM21 and CTD-ILD, but the role of TRIM21 in other lung tissue constituents remains an unsolved mystery and needs to be further investigated. Furthermore, TRIM21 maintains the anti-inflammatory characteristics of lung endothelial cells, potentially contributing to the proof of a link between anti-TRIM21 and CTD-ILD. However, the function of TRIM21 in additional lung tissue elements is still a mystery and requires more research.

Anti-TRIM21 has a high rate of seropositivity for autoantibodies in patients with CTD, and preclinical research indicates its potential role in CTD progression. Nonetheless, the anti-TRIM21 may yield positive results in serum samples from individuals with different infections or tumors. Consequently, its limited specificity has sparked clinical debate regarding its effectiveness as a diagnostic biomarker for CTD-ILD. Current clinical evidence suggests that the presence of anti-TRIM21 antibodies is associated with an increased incidence of concomitant ILD in SjS, SSc, and IIM, with a higher probability of ILD occurring and being more severe, usually predicting poorer outcomes and survival. Intriguingly, most of the relevant published studies are retrospective, and retrospective studies suffer from flaws such as potential bias, impact of treatment, and the lack of adjustment for person-years at risk. Moreover, anti-TRIM21 antibody detection is mostly qualitative or semi-quantitative, and the actual level of autoantibodies may be another important factor in diagnosing the probability of occurrence of CTD-ILD. The above influencing factors limit our ability to correctly assess the diagnostic value of anti-TRIM21 in CTD-ILD. Therefore, more studies are needed to verify whether anti-TRIM21 is a major biological marker for assessing the probability of CTD-ILD occurrence. Nonetheless, the available studies appear to be persuasive that patients with CTD who tests positive for anti-TRIM21 have a higher probability of concurrent ILD, and a high titer of anti-TRIM21 correlates with worse outcomes and shorter overall survival in CTD-ILD patients. However, a more detailed investigation into the connection between anti-TRIM21 and CTD-ILD is required.

Furthermore, the reason why anti-TRIM21 antibodies target the lungs rather than other organs and the significant variability among CTD-ILD patients, seems to suggest a link to immunogenetic factors. Therefore, the influence of genetic factors on TRIM21 and anti-TRIM21 remains a question to be explored.

In general, there are very limited preclinical studies on TRIM21 and anti-TRIM21 antibodies in CTD and CTD-ILD, mostly published data from clinical cohort studies. In this review, we attempt to speculate on the potential molecular mechanisms of TRIM21 and anti-TRIM21 antibodies in CTD-ILD based on the very limited existing foundational research. However, CTD-ILD affects multiple organs and involves complex interactions between molecules, posing significant challenges for studying TRIM21 in CTD-ILD. Therefore, unraveling the molecular mechanisms of TRIM21 in the pathogenesis of CTD-ILD still has a long way to go.

## Author contributions

XG: Conceptualization, Investigation, Methodology, Visualization, Writing – original draft. SH: Investigation, Writing – review & editing, Methodology, Resources. PC: Supervision, Writing – review & editing.
